# Canid hybridization: contemporary evolution in human-modified landscapes

**DOI:** 10.1002/ece3.335

**Published:** 2012-07-25

**Authors:** Astrid V Stronen, Nathalie Tessier, Hélène Jolicoeur, Paul C Paquet, Michel Hénault, Mario Villemure, Brent R Patterson, Tim Sallows, Gloria Goulet, François-Joseph Lapointe

**Affiliations:** 1 Département de sciences biologiques, Université de MontréalMontréal, Québec, H3C 3J7, Canada; 2 Ministère des Ressources naturelles et de la FauneQuébec, Québec, G1S 4X4, Canada (retired); 3 Raincoast Conservation FoundationDenny Island, BC V0T 1B0, Canada; 4 Ministère des Ressources naturelles et de la FauneMont-Laurier, Québec, J9L 2Z9, Canada; 5 Parc national de la MauricieSt-Mathieu-du-Parc, Québec, G0X 1N0, Canada; 6 Ontario Ministry of Natural Resources, Wildlife Research and Development Section, Trent UniversityPeterborough, Ontario, K9J 7B8, Canada; 7 Riding Mountain National ParkWasagaming, Manitoba, R0J 1N0, Canada; 8 Canadian Wildlife Service, Environment CanadaWinnipeg, Manitoba, R3C 4W2, Canada (retired)

**Keywords:** Allopatry, *
Canis*, coyote, hybridization, sympatry, wolf

## Abstract

Contemporary evolution through human-induced hybridization occurs throughout the taxonomic range. Formerly allopatric species appear especially susceptible to hybridization. Consequently, hybridization is expected to be more common in regions with recent sympatry owing to human activity than in areas of historical range overlap. Coyotes (*Canis latrans*) and gray wolves (*C. lupus*) are historically sympatric in western North America. Following European settlement gray wolf range contracted, whereas coyote range expanded to include eastern North America. Furthermore, wolves with New World (NW) mitochondrial DNA (mtDNA) haplotypes now extend from Manitoba to Québec in Canada and hybridize with gray wolves and coyotes. Using mtDNA and 12 microsatellite markers, we evaluated levels of wolf-coyote hybridization in regions where coyotes were present (the Canadian Prairies, *n* = 109 samples) and absent historically (Québec, *n* = 154). Wolves with NW mtDNA extended from central Saskatchewan (51°N, 69°W) to northeastern Québec (54°N, 108°W). On the Prairies, 6.3% of coyotes and 9.2% of wolves had genetic profiles suggesting wolf-coyote hybridization. In contrast, 12.6% of coyotes and 37.4% of wolves in Québec had profiles indicating hybrid origin. Wolves with NW and Old World (*C. lupus*) mtDNA appear to form integrated populations in both regions. Our results suggest that hybridization is more frequent in historically allopatric populations. Range shifts, now expected across taxa following climate change and other human influence on the environment, might therefore promote contemporary evolution by hybridization.

## Introduction

Landscape changes such as urbanization and agricultural development can cause rapid directional selection within species (Reznick et al. 2008) and hybridization because of reduced environmental heterogeneity and collapse of ecological niches (Seehausen et al. 2008). Contemporary evolution through human-induced hybridization has been documented in insects, birds, fish, mammals, and plants (Rhymer and Simberloff 1996; Allendorf et al. 2001; Stockwell et al. 2003).

Gene flow can act as a creative force through the spread of novel genes (Slatkin 1987). Understanding the probability of, and ability for, adaptation in wildlife populations in response to current landscape changes is thus increasingly important to preserve the genetic integrity of wild species (Carroll 2008). The cohesion species-concept (Templeton 1989) highlights the shared importance of genetic exchangeability (gene flow) and ecological exchangeability (shared ecological niche) between organisms. Formerly allopatric species may lack pre-zygotic barriers to reproduction, as reinforcement would not have had the opportunity to evolve, and thus experience increased risk of hybridization (Crispo et al. 2011). Consequently, hybridization is expected to be more frequent in regions with recent sympatry owing to human activity than in areas of historical range overlap.

Species from the genus *Canis* (canids) are distributed across an extensive geographic area (Kurtén and Anderson 1980). Before European settlement, coyotes (*C. latrans*) were considered a western North American species with a range that extended eastward to Manitoba in Canada and Minnesota in the United States (Young and Jackson 1951). Gray wolves (*C. lupus*) historically occurred throughout most of North America outside the southeastern US (Nowak 1995). Sympatric gray wolves and coyotes typically occupy divergent ecological niches (Paquet 1992) without hybridization (e.g., Pilgrim et al. 1998). Gray wolves are considered to have evolved in Eurasia (Kurtén and Anderson 1980) and, hence, to have Old World (OW) mitochondrial DNA (mtDNA).

In eastern North America, wolves with New World (NW) mtDNA have been referred to as eastern wolves, and given species or subspecies status (*C. lycaon* Shreber, 1775 (see also Wilson et al. 2000) or *C. lupus lycaon* (Goldman 1937)). Wolves with NW mtDNA hybridize with gray wolves and coyotes (Koblmüller et al. 2009; Wheeldon and White 2009; Wilson et al. 2009; Fain et al. 2010; Rutledge et al. 2010a; Wheeldon et al. 2010; vonHoldt et al. 2011) and thus seem able to bridge gene flow between canid species. NW mtDNA is common in wolves of the western Great Lakes region, where wolves with NW and OW mtDNA frequently interbreed but wolf-coyote hybridization appears to be rare (Fain et al. 2010; Wheeldon et al. 2010; but see Koblmüller et al. 2009). Western Great Lakes region wolves have been proposed as a separate ecotype (Koblmüller et al. 2009). The evolutionary history and distribution of wolves with NW mtDNA remain uncertain. Their current range in Canada is known to extend from the province of Manitoba in the interior to Québec in the east. The extent of interbreeding between coyotes, wolves with NW mtDNA, and wolves with OW mtDNA across this range is not well understood, and could be influenced by historic differences in range overlap and opportunities for development of ecological and behavioral factors limiting hybridization.

Whereas coyotes benefited greatly from agricultural expansion and have extended their range (Gier 1975), gray wolves experienced extensive range contraction, primarily due to hunting, poisoning, and reduced abundance of large ungulate prey species (Paquet and Carbyn 2003; Leonard et al. 2005). The coyote range expanded eastward and coyotes were observed in southwestern Ontario as early as 1919 (Hilton 1978 and references therein). Agricultural development may have promoted canid hybridization (Lehman et al. 1991; Kyle et al. 2006; Leonard and Wayne 2008). The morphology of hybrid canids reported from agricultural landscapes in southern Ontario seems consistent with selection based on the size and distribution of available prey species (Kolenosky and Stanfield 1975; Schmitz and Lavigne 1987; Sears et al. 2003). The first recorded coyote observation in Québec occurred in Gatineau (northeast of Ottawa) in 1944 (Young and Jackson 1951; Georges 1976). During the mid 1990s, coyotes were reported to have reached west-central Québec (S. Beaudet in Jolicoeur and Hénault 2002, see Note S1 for Québec details).

Predators are greatly susceptible to local extinction (Reznick et al. 2008). Canids show high behavioral flexibility in food acquisition, but are often affected by negative attitudes and persecution (Gier 1975; Fritts and Carbyn 1995; Weaver et al. 1996). In 2001, the Committee on the Status of Endangered Wildlife in Canada designated the eastern wolf (*C. l. lycaon*) a Species of Concern (COSEWIC 2001). The Province of Ontario has provided a similar designation for eastern wolves extending from the Algonquin Provincial Park (hereafter Algonquin) region westward to Lake Superior (Ontario Ministry of Natural Resources 2010).

Our primary purpose was to contrast levels of hybridization in wolves and coyotes from regions with a history of sympatry or parapatry (the Canadian Prairie provinces of Manitoba and Saskatchewan) and allopatry (Québec). We expected hybridization to be infrequent on the Prairies and more common in Québec. Subsequently, we compared areas within the allopatric region that have relatively well-known records of coyote colonization (southern vs. west-central Québec). We anticipated southern Québec canids to form a hybrid swarm, whereas west-central Québec was expected to be an active hybrid zone with greater genetic differentiation between canid types.

## Materials and Methods

### Sampling and study area

We obtained tissue, blood, and hair samples of wolves and coyotes collected during 1990–2010. Samples were contributions from hunters, trappers, and collected from animals captured for radio collaring during other research projects. The samples originated from across Canada with most being collected in Québec and Manitoba (see Table S1 and Fig. S1). Canids were classified as wolf, coyote, or possible canid hybrid according to body mass and morphology, and seven Québec canids were suspected to be hybrids (H. Jolicoeur, unpubl. data). One possible hybrid was identified in Manitoba (V. Crichton, pers. comm.).

We identified the Level II ecoregion of origin for each sample according to the United States Environmental Protection Agency (US EPA 2006). Ecological and climate information on ecoregions are available from the US EPA (2011). Although we do not expect any direct effect of ecoregion on the extent of hybridization, the classification provides a means to (1) describe our overall study area and (2) delineate and compare Québec regions colonized by coyotes at different times. Most Prairie samples were collected in the Riding Mountain National Park (RMNP) region (51°N, 100°W) of Manitoba, representing the intersection between the Temperate Prairies and the Boreal Plain ecoregions (US EPA 2006). Agriculture is the primary landscape modification on the Prairies. Most Québec samples were collected in the Atlantic Highlands, Mixed Wood Plains, Mixed Wood Shield, and Softwood Shield ecoregions. A combination of agriculture and residential development has transformed the landscape throughout the Atlantic Highlands and the Mixed Wood Plains. Residential and industrial-scale forest developments have been the primary changes for the Mixed Wood Shield and the Softwood Shield, although agriculture is present in the Abitibi-Témiscamingue region in west-central Québec near the Ontario border. We also included wolf samples from northern Québec outside the coyote range. These samples were collected in the Hudson Plain and Taiga Shield ecoregions, which have experienced relatively limited landscape development. We included samples from Ontario; wolves from Algonquin (*n* = 34 blood samples), northeastern Ontario (*n* = 6 tissues), and northwestern Ontario (*n* = 6 tissues), coyotes from southern Ontario (*n* = 8 blood samples), and *n* = 6 tissue samples from western Canada for comparison (Table S1).

### DNA extraction, amplification, and genotyping

We extracted DNA using a standard phenol-chloroform protocol, Quicklysis (Olsen et al. 1996), or the PureLink Genomic DNA Mini Kit (Invitrogen Inc., Burlington, Canada) following the mammalian tissue protocol. First, we amplified a portion of the ATP-8 gene of the mtDNA for each individual to classify mtDNA sequences according to Old World (Eurasia) or New World (North America) origins. Here we used primers from Johnson et al. (1998) that amplify a standard band of 150 base pairs (bp) for NW and OW canid mtDNA, and a third primer that amplifies a second band of 100 bp for OW canid mtDNA (N. Tessier, unpubl. data). Amplifications were performed in a multiplex reaction, in a 10 *μ*L volume containing genomic template DNA (100–250 ng), 1× reaction buffer, 2 mmol/L MgCl_2_, 0.25 mmol/L dNTP, 0.5 *μ*mol/L of each primer, and 0.5 U *Taq* polymerase.

Subsequently, we amplified a panel of 11 autosomal and one Y-chromosome microsatellite markers for individuals for whom ATP-8 amplification was successful. Autosomal markers amplified were PEZ15, PEZ19 (Halverson J. in Neff et al. 1999), FH2001, FH2422 (Breen et al. 2001), cxx20, cxx109, cxx172, cxx204, cxx225, cxx250, cxx377 (Ostrander et al. 1993), and Y-chromosome marker MS41A (Sundquist et al. 2001). Amplifications were performed using Polymerase Chain Reaction (PCR) in a 18 *μ*L volume of genomic template DNA (100–250 ng per sample), 1× reaction buffer, 2 mmol/L MgCl_2_, 0.25 mmol/L dNTP, 0.5 *μ*mol/L of primer R, 0.25 *μ*mol/L of primer F labelled with a tail of M13, 0.5 *μ*mol/L of M13-F tail labelled with fluorescent dye HEX or FAM, and 0.5 U *Taq* polymerase.

We amplified DNA using two PCR programs. Conditions were as follows: (1) initial denaturation at 94°C for 15 min, then 38 cycles of 94°C for 30 sec, 58°C for 90 sec, and 72°C for 60 sec, with a final extension at 72°C for 30 min (ATP-8, PEZ15, PEZ19, FH2001, FH2422, MS41A) and (2) initial denaturation at 94°C for 60 sec, then 35 cycles of 94°C for 60 s, 52°C (cxx20, cxx109, cxx204, cxx377) or 58°C (cxx172, cxx225, cxx250) for 45 sec, and 74°C for 60 sec, with a final extension at 72°C for 5 min. We subsequently ran PCR products stained with Bromophenol Blue and SYBR-green (Invitrogen Inc.) on a 3% agarose gel and determined amplification success using UV light.

To compare mtDNA haplotypes in parts of Saskatchewan and Québec with previous findings, 24 samples were sequenced using a portion of the mtDNA control region and primers Thr-L 15926 and DL-H 16340 (Vilà et al. 1999). Amplifications were performed in a 50 *μ*L volume using the product concentrations and PCR conditions described above for the ATP-8 reaction. Genotyping and sequencing was performed using an ABI 3730xl automated sequencer supplied by Applied Biosystems (Life Technologies Inc., Burlington, Ontario, Canada). Microsatellite alleles were scored using GeneMarker1.71 (SoftGenetics, LLC, State College, Pennsylvania). Each allele was scored three independent times and we reamplified and reanalysed 12% of the alleles to confirm the observed genotypes. Control-region mtDNA sequences (387–404 bp) were edited in CLC Sequence Viewer v.6 (http://www.clcbio.com/index.php?id=28) and compared to sequences previously published in GenBank (Table S2).

### Statistical analyses

Samples that were successfully genotyped for at least 9 of 12 markers (for a similar ratio see Pilot et al. 2006) were retained for analyses. We calculated the presence of false alleles and allelic dropout as outlined in Broquet and Petit (2004). Subsequently, we calculated genetic diversity measures (average number of alleles per locus, observed and expected heterozygosity, and F_IS_ with a 95% confidence interval by bootstrapping [*n* = 1000]) using GENETIX4.0 (Belkhir et al. 2004). We determined pairwise population differentiation for coyotes and wolves from Québec and the Prairies by the F_ST_ analogue Theta (Weir and Cockerham 1984) in GENETIX using a test of 999 permutations.

We examined genetic population structure using STRUCTURE 2.3 (Pritchard et al. 2000), a Bayesian clustering program that does not require a priori definition of clusters. We analyzed data for a number of genetic clusters K ranging from 1 to 10 using the admixture model, assuming correlated allele frequencies, and inferring alpha. We ran five repetitions for each K for 10^6^ iterations after burn-in of 10^5^. We assessed the probability for each K-value by calculating the average value over the five repetitions and determined the number of genetic clusters using the values of LnP(D) (equivalent to L(K), Pritchard et al. 2000) and ΔK (Evanno et al. 2005). Thereafter, we ran STRUCTURE 10 times at K = 4 using 10^6^ iterations and a burn-in period of 10^5^, and obtained individual ancestry assignments (i.e., q-values) from the run with the highest probability and the lowest variance (e.g., Fain et al. 2010), including 90% confidence intervals for cluster memberships (q_i_).

**Figure 1:**
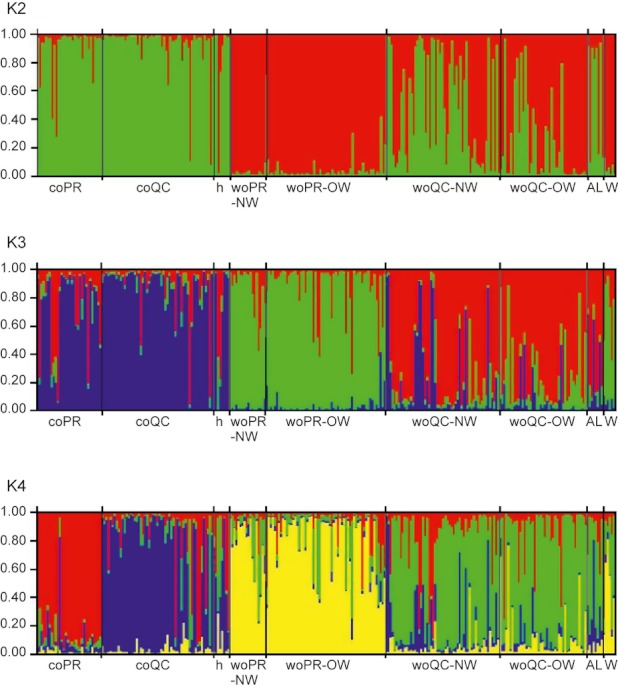
Assignment values for Prairie coyotes (coPR), Québec coyotes (coQC), canids morphologically classified as possible hybrids (h), Prairie wolves (woPR) and Québec wolves (woQC) based on K = 2–4 clusters in STRUCTURE. Wolves are classified as having New World (NW) or Old World (OW) mtDNA. Samples from Algonquin Provincial Park (AL) in Ontario and samples from Western Canada (W) originating from British Columbia, Alberta, and Yukon Territory were included for reference.

We did preliminary STRUCTURE analyses with *n* = 300 canids (*n* = 32 Prairie coyotes, *n* = 55 Québec coyotes, *n* = 8 possible hybrids (Québec and Prairie), *n* = 77 Prairie wolves,^1^ *n* = 99 Québec wolves, *n* = 8 Algonquin wolves, *n* = 6 northeast Ontario wolves, *n* = 6 northwest Ontario wolves, *n* = 3 southern Ontario coyotes, and *n* = 6 Western Canadian wolves. Subsequently, we excluded some reference groups with ≤6 samples to clarify cluster representation (northwest and northeast Ontario wolves and southern Ontario coyotes) and performed analyses using *n* = 285 samples. STRUCTURE results showed that L(K) continued to increase with K, whereas the peak value for ΔK occurred at K = 2 (Table S3). The K = 2 assignment (Fig. 1) generally corresponded with the separation between wolves and coyotes. K = 3 groups split the wolves into two groups, suggesting that Québec and Prairie wolves are more differentiated than are Québec and Prairie coyotes. K = 4 indicated that Québec coyotes, Québec wolves, Prairie coyotes, and Prairie wolves form separate clusters, and we used these results to determine the ancestry of individual canids.

The optimal q-threshold in hybrid studies depends on the aim of the study and requires a trade-off between accuracy and efficiency (Vähä and Primmer 2006). For this reason we used two different thresholds (q = 0.75 and q = 0.90) to identify putative hybrid individuals. Wheeldon et al. (2010) assigned western Great Lakes region canids with q < 0.8 as admixed, whereas Fain et al. (2010) used the value of q < 0.7. We therefore assigned individuals to STRUCTURE clusters using a threshold of q ≥ 0.75. Because our purpose was to detect wolf-coyote hybridization, we classified individuals with Prairie and/or Québec coyote ancestry of q > 0.75 as coyotes, whereas individuals with Prairies and/or Québec wolf ancestry of q ≥ 0.75 were classified as wolves. All other canids were designated as admixed (i.e., wolf-coyote hybrids). Thereafter, we used HYBRIDLAB 1.0 (Nielsen et al. 2006) to evaluate the ability of STRUCTURE to identify hybrid canids among our samples. We selected canids assigned to K = 4 genetic clusters according to a q ≥ 0.90 threshold, where 90% confidence intervals excluded membership in alternate clusters. Subsequently, we simulated parental groups, F1-and F2-hybrids, and back-crossed individuals (a cross between a F1 and a canid from a parental group) with *n* = 30 individuals per group. We evaluated these genotypes in STRUCTURE using the above-mentioned parameters and K = 4 clusters.

Finally, we compared southern Québec wolves with reference samples from neighboring Algonquin and northeastern Ontario populations using principal coordinate analysis (labelled PCA) in GenAlEx (Genetic Analyses in Excel) v.6 (Peakall and Smouse 2006) to evaluate whether wolves in the two areas may have similar profiles.

## Results

### Distribution of NW and OW mtDNA

All coyotes had NW mtDNA. Wolves with NW mtDNA extended from the Manicouagan Reservoir in northeastern Québec (51°N, 69°W; Fig. S1) to Chitek Lake in central Saskatchewan (54°N, 108°W). The wolves from northeastern Québec and central Saskatchewan with NW mtDNA were reported to display morphology and body mass typical of gray wolves (e.g., a 43 kg male in northeastern Québec). NW and OW mtDNA were found in wolves across all ecoregions examined, except in Québec in the Atlantic Highlands (*n* = 2, both NW mtDNA), the Mixed Wood Plains (*n* = 2, both OW mtDNA), the Hudson Plains (*n* = 5, all OW mtDNA) and Taiga Shield (*n* = 7, all OW mtDNA). In Saskatchewan, we found OW and NW mtDNA on both sides of Prince Albert National Park (PANP; 54°N, 106°W). However, *n* = 25 fecal samples (likely from two wolf packs) excluded from our final data set owing to low microsatellite genotyping success showed only OW mtDNA in PANP. Eight of the 11 Manitoba wolves with NW mtDNA were found in and around Duck Mountain Provincial Park and Forest (52°N, 101°W, Fig. S1) and the other three were found in the agricultural landscape surrounding RMNP 30 km to the south. However, only OW mtDNA was found within RMNP.

### mtDNA haplotypes

Wolves from central Saskatchewan had NW and OW mtDNA haplotypes (Table S2); we found NW haplotypes C3 (*n* = 6) and C13 (*n* = 1), and OW haplotype 16 (*n* = 2). One Saskatchewan coyote sequence was shorter than NW haplotype la31, but identical starting from bp 42. Wolves in northeastern Québec had OW haplotype C22 (*n* = 2), and NW haplotype C19 (*n* = 1). A coyote from Îles de la Madeleine (47°N, 62°W) also showed haplotype C19. Elsewhere in Québec (Table S2) we found OW haplotype C22 (*n* = 3) and NW haplotype C14 (*n* = 3) in wolves. In Québec coyotes we identified NW haplotypes C19 (*n* = 1) and C1 (*n* = 1). Two Québec coyotes also had a haplotype shorter than la18 but identical starting from bp 18.

### Genetic diversity and extent of hybridization

For the 300 samples amplified successfully at nine or more microsatellite markers, we found the rate of allelic dropout and false alleles to be 3.2% and 2.8%, respectively. Genetic diversity was high for wolves and coyotes (Table 1). Overall F_ST_ values between wolves and coyotes (Table 2) indicated moderate differentiation (F_ST_ = 0.056–0.121), and similar values were seen between Québec and Prairie wolves (F_ST_ = 0.048–0.072). We found low differentiation between wolves with NW and OW mtDNA both in Québec (F_ST_ = 0.020) and on the Prairies (F_ST_ = 0.025). We obtained successful profiles from all Ontario tissue samples, but only three from southeastern Ontario coyotes and eight from Algonquin wolves. We found OW and NW alleles (Hailer and Leonard 2008) at the MS41A Y-chromosome locus for Québec coyotes, and for Québec wolves with NW and OW mtDNA (Table S1). All Prairie coyotes examined had NW Y-chromosome alleles. Only one Prairie wolf with OW mtDNA had a NW Y-chromosome allele, whereas wolves with NW mtDNA had a similar number of OW and NW Y-chromosome alleles.

**Table 1:** Genetic diversity values for Prairie coyotes (coPR), Québec coyotes (coQC), Prairie wolves (woPR) and Québec wolves (woQC) in Canada. Canids are identified as wolf or coyote according to morphology. Wolves are grouped as having New World (NW) or Old World (OW) mitochondrial DNA (mtDNA)

Group (n)	Average no. alleles/locus	Ho (SE)	He (n.b.)^1^ (SE)	Fis (95% CI by bootstrap, *n* = 1000)
coPR (32)	9.7	0.710 (0.241)	0.739 (0.244)	0.040 (−0.307 to 0.076)
coQC (55)	9.2	0.654 (0.118)	0.717 (0.145)	0.088 (0.032–0.122)
woPR-NW (18)	5.5	0.687 (0.122)	0.699 (0.115)	0.019 (−0.147 to 0.114)
woPR-OW (58)	8.2	0.669 (0.091)	0.707 (0.095)	0.054 (−0.004 to 0.091)
woQC-NW (56)	9.5	0.643 (0.173)	0.705 (0.159)	0.088 (0.031–0.122)
woQC-OW (43)	8.4	0.665 (0.141)	0.719 (0.120)	0.076 (0.007–0.117)

1 Heterozygosity values are calculated with correction for sample size bias (Nei 1978).

SE, standard error.

**Table 2:** Genetic differentiation (F_ST_) with 95% confidence interval estimated by 1000 bootstraps across loci for Prairie coyotes (coPR), Québec coyotes (coQC), Prairie wolves (woPR) and Québec wolves (woQC) in Canada. Canids are identified as wolf or coyote according to morphology. Wolves are grouped as having New World (NW) or Old World (OW) mitochondrial DNA (mtDNA)

	coQC (*n* = 55)	woPR-NW (*n* = 18)	woPR-OW (*n* = 58)	woQC-NW (*n* = 56)	woQC-OW (*n* = 43)
coPR (*n* = 32)	0.062 (0.043–0.080)	0.121 (0.061–0.203)	0.101 (0.057–0.157)	0.065 (0.028–0.106)	0.081 (0.047–0.120)
coQC (*n* = 55)	–	0.111 (0.079–0.145)	0.099 (0.067–0.135)	0.056 (0.035–0.074)	0.081 (0.053–0.109)
woPR-NW (*n* = 18)	–	–	0.025 (0.009–0.041)	0.072 (0.047–0.102)	0.048 (0.026–0.069)
woPR-OW (*n* = 58)	–	–	–	0.064 (0.049–0.080)	0.049 (0.030–0.069)
woQC-NW (*n* = 56)	–	–	–	–	0.020 (0.010–0.031)

We found no obvious differences in microsatellite profiles between wolves with NW and OW mtDNA in either Québec or the Prairies, which is consistent with the F_ST_ results. K = 5 (Fig. S2) suggested that wolves from the Hudson Plains and Taiga Shield ecoregions in Northern Québec (all with OW mtDNA) cluster with certain wolves with NW haplotypes sampled in the Boreal Plain ecoregion of Saskatchewan.

**Figure 2:**
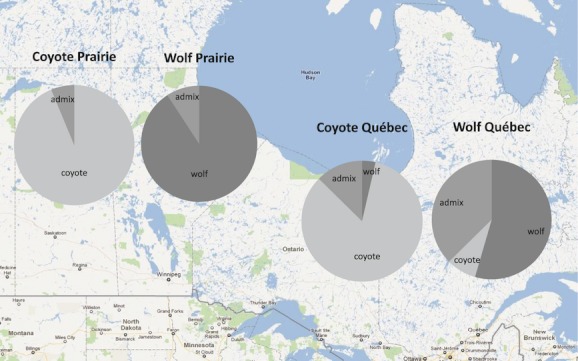
Distribution of canids classified as wolves, coyotes, and admixed individuals on the Prairies and in Québec, Canada, according to STRUCTURE results for K = 4 clusters. Individuals with coyote ancestry of q ≥ 0.75 (from the Prairies and/or Québec) are considered as coyotes, whereas individuals with wolf ancestry of q ≥ 0.75 (Prairies and/or Québec) are classified as wolves.

Individual assignment results from STRUCTURE indicated that hybridization is more frequent in Québec than on the Prairies (Fig. 2). On the Prairies 6.3% of coyotes and 9.2% of wolves had genetic profiles suggesting wolf-coyote hybridization. In contrast, 12.6% of coyotes and 37.4% of wolves in Québec had profiles indicating hybrid origin. Moreover, 3.6% of Québec coyotes were assigned to wolves; one female with unknown morphology and one female with a skull size similar to small wolves (H. Jolicoeur, unpubl. data). In addition, 8.1% of wolves were classified as coyotes. One was assigned to Prairie coyotes (morphology unknown) and two were assigned to Québec coyotes (skulls similar to small wolves, H. Jolicoeur, unpubl. data). Five individuals had combined Québec and Prairie coyote ancestry of q > 0.75. Three had skulls similar to wolves, although one skull was relatively small (H. Jolicoeur, unpubl. data). The morphology of the remaining two canids is unknown. Of eight canids suspected to be hybrids based on morphology, five individuals from Québec and a single Manitoba individual were classified as coyotes. Of the remaining two suspected hybrids, one Québec canid was assigned to wolves and another was identified as admixed. Two of the eight Algonquin wolf profiles suggested affinity to coyotes and six appeared similar to certain wolf profiles from southern Québec.

HYBRIDLAB simulations using the K = 4 STRUCTURE results indicated that most parental genotypes were assigned to the correct group (Table 3). Percentage assignment to the correct group was higher for Prairie wolves (90%) and Québec coyotes (93%) than for Québec wolves (83%) and Prairie coyotes (73%). Correct identification of F1-individuals ranged from 27% to 67%. For selected groups we also simulated F2-individuals, and 33–40% was correctly identified. The proportion of back-crossed individuals placed in the correct group ranged from 7% to 50%.

**Table 3:** HYBRIDLAB simulations using genotypes assigned (q ≥ 0.90) as Prairie wolves (woPR, *n* = 31), Québec coyotes (coQC, *n* = 31), Québec wolves (woQC, *n* = 23), Prairie coyotes (coPR, *n* = 22). *N* = 30 genotypes were simulated for each group of parental, F1, F2, and backcross genotypes

Group	Classification	Composition	Percent correct assignment	Notes on incorrect assignment
1	Parental	woPR	90	
2	Parental	coQC	93	
3	Parental	woQC	83	
4	Parental	coPR	73	
5	F1	woPR × coPR	27	37% to woPR
6	F1	woPR × coQC	57	23% to coQC
7	F1	woPR × woQC	67	20% to woPR
8	F1	coQC × woQC	53	33% to coQC
9	F1	coQC × coPR	30	20% to coQC
10	F1	woQC × coPR	33	40% to coPR
11	F2^1^	woPR × coPR	33	47% to woPR
12	F2	woQC × coPR	40	53% to coPR
13	F2	woQC × coQC	40	33% to coQC
14	Backcross^2^	woPR × (woPR × coPR)	10	80% to woPR
15	Backcross	coPR × (woPR × coPR)	30	40% to coPR
16	Backcross	woQC × (woQC × coQC)	50	37% to woQC
17	Backcross	coQC × (woQC × coQC)	7	83% to coQC
18	Backcross	woQC × (woQC × coPR)	30	47% to woQC
19	Backcross	coQC × (woQC × coPR)	20	57% to coQC

1 F1 × F1.

2 F1 × parental genotype.

The STRUCTURE results for Québec canids are organized in a south to north order for each group (Fig. 1). The findings appear to support our expectation of detecting both a higher proportion of hybrids and a higher variability in genetic profiles toward the northern extent of the coyote range. Northern Québec coyotes have more diverse profiles than those found in southern Québec, and several individuals show high affinity to either Prairie coyotes or Québec wolves. Two coyotes from western Québec were assigned to wolves and eight wolves with NW mtDNA (six from western Québec and two from south of the St. Lawrence River) were assigned to coyotes. Only wolves with OW mtDNA were found in the Hudson Plains and Taiga Shield ecoregions in northern Québec. None of the wolves with OW mtDNA was assigned to coyotes.

PCA analyses suggested that the overlap between coyote and wolf profiles might be higher in the Mixed Wood Shield ecoregion (Fig. S3) than in the Softwood Shield (Fig. S4). Mixed Wood Shield results indicated high overlap between eight wolves from Algonquin and southern Québec wolves, whereas Softwood Shield results showed limited overlap between six northeastern Ontario wolves and neighboring Québec wolves. Overall, analyses of Algonquin and Québec canids (Fig. S5) suggested limited overlap between Algonquin wolves and Québec coyotes, which appears consistent with the STRUCTURE results.

## Discussion

### mtDNA haplotype distribution

These results are, to our knowledge, the first reports of Saskatchewan and northeastern Québec wolves with NW mtDNA, although a similar Québec range extension for *C. l. lycaon* was indicated by Nowak (1983, 1995). The Saskatchewan findings were expected, as similar haplotypes occur in western Manitoba (Wilson et al. 2000; Stronen et al. 2010). Previous studies have indicated genetic structuring between protected areas and their surroundings, with OW mtDNA being more common within Pukaskwa National Park in Ontario (Wilson et al. 2009) and RMNP (Stronen et al. 2010). Although we found only 18 Prairie wolves with NW mtDNA, their distribution is consistent with previous findings (Stronen et al. 2010). The possibility of genetic structuring between areas such as PANP (see also Urton 2004) and their surroundings merits future investigation, and should examine the role of protected areas in preserving wolf social structure (Rutledge et al. 2010b) for range edge populations.

The Québec mtDNA results seem to concur with earlier findings from eastern Ontario and southwestern Québec (e.g., Lehman et al. 1991; Wilson et al. 2000, 2009; Grewal et al. 2004; Rutledge et al. 2010a) and the northeastern US (Kays et al. 2010; Bozarth et al. 2011). The large male from northeastern Québec with haplotype C19 nevertheless extends the range of wolves with NW mtDNA approximately 400 km from the nearest reported location near Lac St-Jean (49°N, 72°W) in east-central Québec. Although C19 is common in Algonquin wolves (Rutledge et al. 2010a) and could have been introduced to Québec via dispersal, this finding raises questions on the extent to which wolf-coyote hybridization might influence wolves in ecosystems farther north than previously thought. The C19 haplotype was shared with a coyote from Havre-Aubert on Îles de la Madeleine (47°N, 62°W) in eastern Québec, and further research should examine the possibility of wolf-coyote gene flow in the northeast. Coyotes occurred in southeastern North America during parts of the Pleistocene, although all known records are >10,000 years old (Nowak 2003). Complete mtDNA-replacement following introgressive hybridization has been documented in several taxa, including reports from our study area (Wilson and Bernatchez 1998). This possibility cannot be excluded for wolves with NW mtDNA, but provides no clear explanation for why contemporary wolf-coyote hybridization appears more common in Québec than on the Prairies.

### Microsatellite genetic diversity and extent of hybridization

The STRUCTURE results for K = 4 showed major genetic structuring between wolves and coyotes. Whereas wolf-coyote hybridization seems relatively rare on the Prairies, it appears common in Québec. These findings correspond with previous results from the western Great Lakes region (hybridization rare; Fain et al. 2010; Wheeldon et al. 2010; but see Koblmüller et al. 2009 and vonHoldt et al. 2011 for an alternate view), southern Ontario (hybridization common; Wilson et al. 2009; Rutledge et al. 2010a), and the northeastern US (hybridization common but reflecting past introgression; Kays et al. 2010; Bozarth et al. 2011; vonHoldt et al. 2011).

Overall genetic diversity was high, and the lower allelic diversity observed for Prairie wolves with NW mtDNA was likely influenced by the smaller sample size. For this reason, the *F*-statistics involving this group should also be interpreted with caution. F_ST_ values between coyotes and wolves were moderate (Balloux and Lugon-Moulin 2002) and might have been affected by sample size and substructuring (Wahlund effect; Wahlund 1928). Overall, F_ST_ results seem consistent with STRUCTURE, including the finding that Québec wolves with NW mtDNA were less differentiated from coyotes than were other wolves. The low differentiation between wolves with NW and OW mtDNA is also in accord with previous reports (Fain et al. 2010; Wheeldon et al. 2010).

The grouping of wolves from northern Québec and several Prairie wolves from central Saskatchewan at K = 5 suggested that gene flow might be higher in northern areas of Canada. Likewise, vonHoldt et al. (2011) reported similarity in SNP profiles for wolves from northern Québec and western North America. The K = 5 cluster comprised wolves with OW mtDNA from Québec and wolves with NW mtDNA from Saskatchewan, which appears further to support the lack of obvious microsatellite genetic structuring between wolves with NW and OW mtDNA. The possibility of such structuring should nonetheless be examined in detail using additional markers (e.g., Godinho et al. 2011). Most canids categorized as possible hybrids according to morphology were assigned to coyotes, although one was assigned to wolves and another was identified as admixed. The apparent difficulties in detecting backcrosses suggest caution in assigning these individuals. Further analyses (including comparison with dog profiles) would be required to elucidate their ancestry.

Northern Québec coyotes seem to include more hybrid individuals (according to microsatellite results) and higher variation in genetic profiles, which could indicate an active hybrid zone. Wolves appear to have been absent from the south shore of the St. Lawrence River in Québec for many years (Banville 1983) although some individuals have been found recently (Villemure and Jolicoeur 2004). Coyotes in this area are thus unlikely to receive (substantial) genetic contributions from wolves at present. Several Algonquin and southern Québec wolves had similar profiles, which accords with an earlier report (Villemure and Jolicoeur 2004) and movements of radio-collared individuals in both directions (M. Villemure and B. Patterson, monitoring data). Importantly, results from a larger sample showed that Algonquin forms a separate cluster (Rutledge et al. 2010a). This was also the case when 38 individuals from southern Québec were included (L. Rutledge, unpubl. data). Additional genomic investigation will be required to resolve canid ancestry and distribution in this region, and to determine the extent to which geographical differences in the frequency of coyote hybridization might be influenced by the presence of different types of wolves.

Our HYBRIDLAB results suggested variable probabilities of correctly identifying hybrids. The results appeared influenced by the sample sizes and identification probability for parental groups. The number of individuals available for simulation of Québec wolves and Prairie coyotes was lower (*n* = 23 and *n* = 22) than for Prairie wolves and Québec coyotes (both *n* = 31). Nielsen et al. (2006) recommended using 30–50 individuals for simulations, but this was not always possible when applying the q ≥ 0.9 assignment criterion. Whereas STRUCTURE assigned 12.6% of coyotes and 37.4% of wolves in Québec as hybrids, HYBRIDLAB simulations suggested low probabilities of correctly identifying backcrosses involving Québec coyotes and Prairie wolves. Our estimate for Québec coyotes may therefore be highly conservative. The finding of OW Y-chromosome alleles in six (of *n* = 16) Québec coyotes further supports wolf-coyote hybridization. Most Québec wolves had OW Y-chromosome alleles, including wolves with NW mtDNA. Although our sample is limited, this suggests that admixture may be asymmetric between male and female canids. As Prairie (and western Great Lakes region) wolf-coyote hybridization seems relatively rare, the significance of our apparent limited ability to identify backcrosses with Prairie wolves is not clear and needs further evaluation. Few Prairie wolves had NW Y-chromosome alleles. However, most of our Prairie samples were collected in the RMNP region, and profiles from this area may not be fully representative for the Canadian Prairies.

Assessments of hybrid canids with known ancestry have demonstrated variable estimates of admixture depending on the software program and criteria used (Bohling and Waits 2011). Randi (2008) also reported difficulties in correctly identifying wolf-dog backcrosses simulated in HYBRIDLAB. Many “pure” (non-hybridizing) coyotes reported from southern Québec could thus have wolf ancestry that is no longer detectable with our panel of markers. Our PCA results appeared to suggest that southern Québec coyotes had progressed toward a hybrid swarm, whereas northern coyotes may represent an active hybrid zone. Results within the current zone of wolf-coyote sympatry were nonetheless ambiguous and supplementary analyses with additional genetic markers and fine-scale sampling are required to delineate active hybrid fronts.

### Ecological and evolutionary implications of hybridization

Wolf-coyote hybridization seems rare where these species were historically sympatric (i.e., Canadian Prairie provinces) and more frequent where they were formerly allopatric (i.e., Québec). Future research could help elucidate why wolves with NW genetic material appear rarely to hybridize with western coyotes but seem well integrated with western wolves. Conversely, westward expansion of hybrids from eastern North America might promote dissolution of existing reproductive barriers, especially if hybridization with coyotes facilitates wolf adaptation to human-modified landscapes (Kyle et al. 2006). Wolves with NW mtDNA may also extend farther to the east and west, which should be assessed in contiguous regions.

In southern Québec, the combination of frequent wolf-coyote hybridization and limited hunting and trapping regulations might threaten the ecological role of wolves. High human-caused mortality can disrupt wolf social structure and promote hybridization with coyotes (Rutledge et al. 2010b). In addition, human harvest has been shown to diminish body size in many species (Darimont et al. 2009). Wolf body size can decline across narrow spatiotemporal scales (Mech and Paul 2008; Stronen et al. 2010), and the presence of smaller wolves might facilitate hybridization with coyotes where environmental heterogeneity, and thus the opportunity for niche divergence, has been reduced (Seehausen et al. 2008). The consequences of harvest could therefore extend beyond the number of individuals removed from areas such as the region surrounding the 536 km^2^ Mauricie National Park (47°N, 73°W), one of the few protected areas for wolves in southern Québec (COSEWIC 2001). Despite two adjoining wildlife reserves, the packs found within (or partially within) the Park borders experience high human-caused mortality (Villemure 2003).

Wolves with NW and OW mtDNA appear to form integrated populations throughout much of southern Québec and the Canadian Prairies, which suggest that they interbreed within a shared ecological niche (Templeton 1989) defined by the historical role of wolves as large ungulate predators. Recent analyses of 48K SNP markers also support an origin of wolves with NW mtDNA predating European settlement (vonHoldt et al. 2011). Conservation efforts should therefore focus on preserving the overall ecological function of wolves and the predator-prey relationships of which they are part. Accordingly, measures to protect the wolf population from coyote hybridization ought to be considered in southern Québec. Human influence might now reverse speciation (Seehausen et al. 2008) and the relationship between ecological divergence and reproductive isolation appears strong across taxa (Funk et al. 2006). A research priority is thus to understand better the extent to which reproductively compatible but ecologically different groups such as coyotes and wolves may coexist without (further) hybridization given the opportunity for divergent prey selection.

## Footnotes

1 Henceforth referring to wolves from the Canadian Prairie provinces, and not to be confused with the term “prairie wolves” at times used as a synonym for coyotes.

## Supporting Information

**Note S1.** Coyote range expansion in Québec.

**Table S1.** Canid samples from Canada used in analyses of wolf-coyote hybridization.

**Table S2.** Mitochondrial DNA (mtDNA) control-region haplotypes in wolves and coyotes from Saskatchewan and Québec, Canada. Sample locations are provided in Table S1.

**Table S3.** Probability values for the number of genetic clusters (L(K) with standard deviation [SD] and ΔK) for wolves and coyotes from the Prairies and Québec, Canada, using K = 1–10.

**Figure S1.** Map of primary sampling locations for the Prairies (Manitoba and Saskatchewan) and Québec, Canada. Ontario reference samples are shown, but six reference samples from Western Canada (British Columbia, Alberta, and Yukon Territory) are not included. Areas where 1–10 samples were collected are marked in red, and areas where 11–50 samples were collected are marked in blue. UTM locations and identification of canids are provided in Table S1. Wolves with NW mtDNA haplotypes extended eastward to Manicouagan Reservoir, Québec (1) and westward to Chitek Lake, Saskatchewan (2). Manitoba wolves with NW mtDNA were located in or near Duck Mountain Provincial Park and Forest (3).

**Figure S2.** Assignment values for Prairie coyotes (coPR), Québec coyotes (coQC), canids morphologically classified as possible hybrids (h), Prairie wolves (woPR) and Québec wolves (woQC) according to STRUCTURE results for K = 5 clusters. Wolves are classified as having New World (NW) or Old World (OW) mtDNA. Samples from Algonquin Provincial Park (AL) in Ontario and samples from Western Canada (W) originating from British Columbia, Alberta, and Yukon Territory were included for reference.

**Figure S3.** Principal coordinate analysis for Québec coyotes (coQC, *n* = 13), wolves with New World (woQC-NW, *n* = 34) and Old World (woQC-OW, *n* = 12) mtDNA, and wolves from Algonquin Provincial Park, Ontario (*n* = 8) sampled in the Mixed Wood Shield ecoregion. The first and second axes explained 22.0% and 19.1% of the variation, respectively.

**Figure S4.** Principal coordinate analysis for Québec coyotes (coQC, *n* = 8), wolves with New World (woQC-NW, *n* = 20) and Old World (woQC-OW, *n* = 17) mtDNA, and wolves from northeastern Ontario (*n* = 6) sampled in the Softwood Shield ecoregion. The first and second axes explained 23.3% and 18.9% of the variation, respectively.

**Figure S5**. Principal coordinate analysis for Québec coyotes (coQC, *n* = 55), wolves with New World (woQC-NW, *n* = 56) and Old World (woQC-OW, *n* = 43) mtDNA, and wolves from Algonquin Provincial Park, Ontario (*n* = 8). The first and second axes explained 25.6% and 19.3% of the variation, respectively.
