# Supplementary material for: Canid hybridization: contemporary evolution in human-modified landscapes
Source: Ecol Evol. 2012 Jul 25;2(9):2128–40. doi: 10.1002/ece3.335 (PMC3488665; doi:10.1002/ece3.335)
Supplement: Supplementary file 1 [file ece30002-2128-SD1.doc]

**Supplemental material for the manuscript ‘Canid hybridization: contemporary evolution in human-modified landscapes’ (Stronen et al. 2012).**

Note S1. Coyote range expansion in Québec

The first recorded coyote observation in Québec occurred in Gatineau northeast of Ottawa in 1944 (Young and Jackson 1951, Georges 1976). By 1963 the coyote range extended throughout the southwestern portion of Québec south of the St. Lawrence River and north to La Malbaie (48N, 70W) on the north shore. Coyotes were subsequently recorded on the Gaspé peninsula (49N, 67W) in 1973 and on the north shore (50N, 63W) of the Gulf of St. Lawrence in 1991 (Larivière and Crête 1992). During the mid 1990s coyotes were reported near Matagami (50N, 78W) in westcentral Québec (S. Beaudet in Jolicoeur and Hénault 2002).

Literature cited:

Georges S (1976) A range extension of the coyote in Quebec. The Canadian Field-Naturalist 90: 78-79.

Jolicoeur H, Hénault M (2002). Répartition géographique du loup et du coyote au sud du 52^e^ parallèle et estimation de la population de loups au Québec. Société de la faune et des parcs du Québec. 41pp. plus annexe.

Larivière S, Crête M (1992) Causes et conséquences de la colonisation du Québec par le coyote (*Canis latrans*). Ministère du Loisir, de la Chasse et de la Pêche, Direction des espèces et des habitats, Québec.

Young SP, Jackson HHT (1951) The clever coyote. The Stockpile Co., Harrisburg, PA, and the Wildlife Management Institute, Washington, D.C.

Table S1. Canid samples from Canada used in analyses of wolf-coyote hybridization.

| **Sample** | **Sex** | **Province^1^** | **Morphology** | **mtDNA^2^** | **Y^3^** | **Ezozone^4^** | **UTM East** | **UTM North** | **UTM zone** |
| --- | --- | --- | --- | --- | --- | --- | --- | --- | --- |
| Co618 | F | QC | coyote | NW |  | Mixed Wood Plains | 587255 | 5232046 | 20 T |
| Co619 | ? | QC | coyote | NW |  | Mixed Wood Plains | 290722 | 5166200 | 19 T |
| Co621 | M | QC | coyote | NW | OW | Atlantic Highlands | 321616 | 5052618 | 19 T |
| Co622 | M | QC | coyote | NW |  | Atlantic Highlands | 321616 | 5052618 | 19 T |
| Co625 | M | QC | coyote | NW | NW | Atlantic Highlands | 683793 | 4989298 | 18 T |
| Co628 | F | QC | coyote | NW |  | Mixed Wood Plains | 316523 | 5174625 | 19 T |
| Co629 | F | QC | coyote | NW |  | Mixed Wood Plains | 318687 | 5153150 | 19 T |
| Co630 | M | QC | coyote | NW | NW | Atlantic Highlands | 356407 | 5080901 | 19 T |
| Co632 | F | QC | coyote | NW |  | Mixed Wood Plains | 290722 | 5166200 | 19 T |
| Co635 | M | QC | coyote | NW | NW | Mixed Wood Plains | 290722 | 5166200 | 19 T |
| Co637 | M | QC | coyote | NW |  | Atlantic Highlands | 410000 | 5200687 | 19 T |
| Co638 | M | QC | coyote | NW | NW | Mixed Wood Plains | 340905 | 5152248 | 19 T |
| Co639 | F | QC | coyote | NW |  | Softwood Shield | 510611 | 5525420 | 18 U |
| Co647 | M | QC | coyote | NW | OW | Mixed Wood Shield | 284094 | 5196896 | 19 T |
| Co648 | F | QC | coyote | NW |  | Mixed Wood Shield | 312238 | 5200706 | 19 T |
| Co649 | M | QC | coyote | NW | OW | Mixed Wood Plains | 290722 | 5166200 | 19 T |
| Co657 | M | QC | coyote | NW |  | Mixed Wood Plains | 302389 | 5171368 | 19 T |
| Co658 | F | QC | coyote | NW |  | Mixed Wood Plains | 316523 | 5174625 | 19 T |
| Co660 | M | QC | coyote | NW |  | Atlantic Highlands | 444303 | 5210981 | 19 T |
| Co663 | F | QC | coyote | NW |  | Atlantic Highlands | 368840 | 5176958 | 19 T |
| Co664 | M | QC | coyote | NW |  | Mixed Wood Plains | 290722 | 5166200 | 19 T |
| Co665 | ? | QC | coyote | NW |  | Atlantic Highlands | 385594 | 5119194 | 19 T |
| Co671 | M | QC | coyote | NW | NW | Mixed Wood Shield | 531406 | 5151285 | 18 T |
| Co676 | F | QC | coyote | NW |  | Atlantic Highlands | 412002 | 5166913 | 19 T |
| Co678 | M | QC | coyote | NW | NW | Mixed Wood Shield | 722791 | 5214897 | 18 T |
| Co679 | F | QC | coyote | NW |  | Mixed Wood Plains | 284860 | 5181248 | 19 T |
| Co697 | F | QC | coyote | NW |  | Mixed Wood Plains | 302389 | 5171368 | 19 T |
| Co699 | F | QC | coyote | NW |  | Mixed Wood Plains | 302389 | 5171368 | 19 T |
| Co703 | F | QC | coyote | NW |  | Mixed Wood Plains | 318687 | 5153150 | 19 T |
| Co705 | M | QC | coyote | NW | NW | Softwood Shield | 287966 | 5313778 | 18 T |
| Co715 | M | QC | coyote | NW |  | Mixed Wood Shield | 651137 | 5142227 | 18 T |
| Co717 | M | QC | coyote | NW | NW | Atlantic Highlands | 286879 | 5016169 | 19 T |
| Co727 | M | QC | coyote | NW |  | Atlantic Highlands | 367028 | 5032492 | 19 T |
| Co731 | M | QC | coyote | NW | OW | Mixed Wood Plains | 316523 | 5174625 | 19 T |
| Co732 | M | QC | coyote | NW | NW | Atlantic Highlands | 338250 | 5029464 | 19 T |
| Co733 | M | QC | coyote | NW | OW | Atlantic Highlands | 268139 | 5005693 | 19 T |
| Co735 | M | QC | coyote | NW |  | Mixed Wood Plains | 302389 | 5171368 | 19 T |
| Co777 | F | QC | coyote | NW |  | Softwood Shield | 366883 | 5466801 | 18 U |
| Co892 | F | QC | coyote | NW |  | Mixed Wood Shield | 517794 | 5144351 | 18 T |
| Co911 | M | QC | coyote | NW |  | Softwood Shield | 645994 | 5345957 | 17 U |
| Co932 | M | QC | coyote | NW |  | Softwood Shield | 645994 | 5345957 | 17 U |
| Co933 | M | QC | coyote | NW |  | Softwood Shield | 645994 | 5345957 | 17 U |
| Co934 | F | QC | coyote | NW |  | Softwood Shield | 698596 | 5328999 | 17 U |
| Co936 | M | QC | coyote | NW |  | Mixed Wood Shield | 626316 | 5225037 | 17 T |
| Co937 | F | QC | coyote | NW |  | Mixed Wood Shield | 635820 | 5251187 | 17 T |
| Co1086 | F | QC | coyote | NW |  | Atlantic Highlands | 325407 | 5054079 | 19 T |
| Co1087 | M | QC | coyote | NW | NW | Atlantic Highlands | 320759 | 5051464 | 19 T |
| Co1088 | M | QC | coyote | NW | OW | Mixed Wood Plains | 694714 | 5054996 | 18 T |
| Co1089 | M | QC | coyote | NW |  | Atlantic Highlands | 320759 | 5051464 | 19 T |
| Co1103 | M | QC | coyote | NW |  | Mixed Wood Shield | 465854 | 5157234 | 18 T |
| Co1106 | F | QC | coyote | NW |  | Mixed Wood Shield | 475057 | 5179413 | 18 T |
| Co1120 | M | QC | coyote | NW |  | Mixed Wood Shield | 466144 | 5132841 | 18 T |
| Co1131 | ? | QC | coyote | NW |  | Softwood Shield | 615161 | 5400849 | 17 U |
| CoMC06-25 | M | QC | coyote | NW |  | Mixed Wood Shield | 664650 | 5165000 | 18 T |
| CoMC01-13 | M | QC | coyote | NW |  | Mixed Wood Shield | 670700 | 5163700 | 18 T |
| Ly530 | M | QC | wolf | NW | OW | Softwood Shield | 304441 | 5380824 | 19 U |
| Ly531 | M | QC | wolf | NW |  | Atlantic Highlands | 321616 | 5052618 | 19 T |
| Ly642 | M | QC | wolf | NW |  | Mixed Wood Shield | 680060 | 5143104 | 17 T |
| Ly643 | F | QC | wolf | NW |  | Softwood Shield | 355086 | 5434909 | 18 U |
| Ly644 | F | QC | wolf | NW |  | Softwood Shield | 355086 | 5434909 | 18 U |
| Ly645 | M | QC | wolf | NW |  | Mixed Wood Shield | 692567 | 5149394 | 17 T |
| Ly656 | ? | QC | wolf | NW |  | Softwood Shield | 690198 | 5393626 | 18 U |
| Ly666 | M | QC | wolf | NW |  | Mixed Wood Shield | 718173 | 5236979 | 18 T |
| Ly667 | ? | QC | wolf | NW |  | Mixed Wood Shield | 546207 | 5229915 | 18 T |
| Ly673 | ? | QC | wolf | NW |  | Mixed Wood Shield | 531406 | 5151285 | 18 T |
| Ly682 | M | QC | wolf | NW | OW | Atlantic Highlands | 327514 | 5018632 | 19 T |
| Ly688 | F | QC | wolf | NW |  | Softwood Shield | 355086 | 5434909 | 18 U |
| Ly690 | F | QC | wolf | NW |  | Mixed Wood Shield | 626615 | 5234520 | 18 T |
| Ly691 | ? | QC | wolf | NW |  | Mixed Wood Shield | 341498 | 5296241 | 19 T |
| Ly700 | M | QC | wolf | NW | OW | Mixed Wood Shield | 307340 | 5216001 | 19 T |
| Ly704 | ? | QC | wolf | NW |  | Softwood Shield | 304441 | 5380824 | 19 U |
| Ly706 | F | QC | wolf | NW |  | Mixed Wood Shield | 305944 | 5273871 | 18 T |
| Ly707 | M | QC | wolf | NW | OW | Mixed Wood Shield | 306238 | 5273884 | 18 T |
| Ly713 | M | QC | wolf | NW |  | Mixed Wood Shield | 294277 | 5213691 | 19 T |
| Ly716 | M | QC | wolf | NW | OW | Softwood Shield | 310313 | 5514163 | 18 U |
| Ly808 | M | QC | wolf | NW | OW | Mixed Wood Shield | 672083 | 5285561 | 18 T |
| Ly825 | M | QC | wolf | NW |  | Mixed Wood Shield | 471104 | 5084460 | 18 T |
| Ly826 | M | QC | wolf | NW | OW | Mixed Wood Shield | 477559 | 5121310 | 18 T |
| Ly830 | F | QC | wolf | NW |  | Mixed Wood Shield | 406265 | 5103161 | 18 T |
| Ly867 | F | QC | wolf | NW |  | Mixed Wood Shield | 624619 | 5245379 | 17 T |
| Ly869 | ? | QC | wolf | NW |  | Softwood Shield | 700643 | 5410683 | 17 U |
| Ly870 | ? | QC | wolf | NW |  | Softwood Shield | 700643 | 5410683 | 17 U |
| Ly871 | ? | QC | wolf | NW |  | Softwood Shield | 305226 | 5367811 | 18 U |
| Ly872 | ? | QC | wolf | NW |  | Softwood Shield | 292789 | 5331162 | 18 U |
| Ly875 | F | QC | wolf | NW |  | Mixed Wood Shield | 331355 | 5290966 | 19 T |
| Ly876 | M | QC | wolf | NW | OW | Mixed Wood Shield | 331355 | 5290966 | 19 T |
| Ly894 | M | QC | wolf | NW |  | Softwood Shield | 322360 | 5402483 | 18 U |
| Ly896 | F | QC | wolf | NW |  | Softwood Shield | 712704 | 5383304 | 17 U |
| Ly899 | M | QC | wolf | NW |  | Softwood Shield | 632180 | 5406786 | 17 U |
| Ly901 | F | QC | wolf | NW |  | Softwood Shield | 334595 | 5359469 | 18 U |
| Ly905 | M | QC | wolf | NW |  | Softwood Shield | 614511 | 5347150 | 17 U |
| Ly914 | F | QC | wolf | NW |  | Softwood Shield | 647274 | 5344133 | 17 U |
| Ly915 | F | QC | wolf | NW |  | Softwood Shield | 625142 | 5447406 | 17 U |
| Ly921 | M | QC | wolf | NW |  | Mixed Wood Shield | 626316 | 5225037 | 17 T |
| Ly926 | F | QC | wolf | NW |  | Softwood Shield | 685278 | 5356367 | 17 U |
| Ly1107 | F | QC | wolf | NW |  | Mixed Wood Shield | 485114 | 5189003 | 18 T |
| Ly1113 | F | QC | wolf | NW |  | Mixed Wood Shield | 436569 | 5162126 | 18 T |
| Ly1118 | M | QC | wolf | NW |  | Mixed Wood Shield | 482483 | 5143706 | 18 T |
| Ly1124 | M | QC | wolf | NW |  | Mixed Wood Shield | 520509 | 5135748 | 18 T |
| Ly1134 | M | QC | wolf | NW |  | Softwood Shield | 491457 | 5646125 | 19 U |
| LyFM01-03 | F | QC | wolf | NW |  | Mixed Wood Shield | 628706 | 5179284 | 18 T |
| LyFM02-04 | F | QC | wolf | NW |  | Mixed Wood Shield | 629291 | 5179447 | 18 T |
| LyMM01-06 | M | QC | wolf | NW |  | Mixed Wood Shield | 629291 | 5179447 | 18 T |
| LyFM04-07 | F | QC | wolf | NW |  | Mixed Wood Shield | 628706 | 5179284 | 18 T |
| LyFW01-09 | F | QC | wolf | NW |  | Mixed Wood Shield | 647119 | 5215715 | 18 T |
| LyFN01-11 | F | QC | wolf | NW |  | Mixed Wood Shield | 647119 | 5215715 | 18 T |
| LyMN02-12 | M | QC | wolf | NW | NW | Mixed Wood Shield | 635622 | 5219899 | 18 T |
| LyMN03-22 | M | QC | wolf | NW | NW | Mixed Wood Shield | 628500 | 5211600 | 18 T |
| LyMM03-27 | M | QC | wolf | NW | OW | Mixed Wood Shield | 639200 | 5186700 | 18 T |
| LyFP03-30 | F | QC | wolf | NW |  | Mixed Wood Shield | 650600 | 5180400 | 18 T |
| LyMI03-32 | M | QC | wolf | NW | NW | Mixed Wood Shield | 670026 | 5158936 | 18 T |
| LuFM05-19 | F | QC | wolf | OW |  | Mixed Wood Shield | 638800 | 5178250 | 18 T |
| Lu3 | ? | QC | wolf | OW |  | Taiga Shield | 393283 | 6436263 | 20 V |
| Lu4 | ? | QC | wolf | OW |  | Taiga Shield | 393283 | 6436263 | 20 V |
| Lu5 | ? | QC | wolf | OW |  | Taiga Shield | 393283 | 6436263 | 20 V |
| Lu6 | ? | QC | wolf | OW |  | Taiga Shield | 495222 | 6469072 | 19 V |
| Lu501 | F | QC | wolf | OW |  | Softwood Shield | 711054 | 5427439 | 17 U |
| Lu527 | ? | QC | wolf | OW |  | Softwood Shield | 310313 | 5514163 | 18 U |
| Lu528 | ? | QC | wolf | OW |  | Softwood Shield | 310313 | 5514163 | 18 U |
| Lu654 | M | QC | wolf | OW |  | Softwood Shield | 679192 | 5430348 | 18 U |
| Lu674 | ? | QC | wolf | OW |  | Mixed Wood Shield | 531406 | 5151285 | 18 T |
| Lu675 | ? | QC | wolf | OW |  | Mixed Wood Shield | 531406 | 5151285 | 18 T |
| Lu692 | ? | QC | wolf | OW |  | Hudson Plain | 328028 | 5851213 | 18 U |
| Lu693 | ? | QC | wolf | OW |  | Hudson Plain | 328028 | 5851213 | 18 U |
| Lu701 | M | QC | wolf | OW | NW | Mixed Wood Shield | 370700 | 5287000 | 19 T |
| Lu708 | M | QC | wolf | OW |  | Mixed Wood Shield | 341498 | 5296241 | 19 T |
| Lu709 | F | QC | wolf | OW |  | Softwood Shield | 614569 | 5552357 | 19 U |
| Lu710 | F | QC | wolf | OW |  | Softwood Shield | 289918 | 5576150 | 20 U |
| Lu712 | M | QC | wolf | OW |  | Mixed Wood Shield | 294277 | 5213691 | 19 T |
| Lu729 | ? | QC | wolf | OW |  | Hudson Plain | 332354 | 5788235 | 18 U |
| Lu730 | ? | QC | wolf | OW |  | Hudson Plain | 334781 | 5803105 | 18 U |
| Lu734 | M | QC | wolf | OW | OW | Mixed Wood Plains | 682403 | 5134733 | 18 T |
| Lu746 | M | QC | wolf | OW | NW | Mixed Wood Plains | 351701 | 5153264 | 19 T |
| Lu813 | F | QC | wolf | OW |  | Mixed Wood Shield | 540971 | 5126273 | 18 T |
| Lu822 | M | QC | wolf | OW | OW | Mixed Wood Shield | 496454 | 5108715 | 18 T |
| Lu823 | M | QC | wolf | OW | OW | Mixed Wood Shield | 536983 | 5128504 | 18 T |
| Lu831 | M | QC | wolf | OW | OW | Softwood Shield | 647274 | 5344133 | 17 U |
| Lu833 | M | QC | wolf | OW | OW | Mixed Wood Shield | 536983 | 5128504 | 18 T |
| Lu838 | F | QC | wolf | OW |  | Mixed Wood Shield | 536983 | 5128504 | 18 T |
| Lu862 | ? | QC | wolf | OW |  | Softwood Shield | 632180 | 5406786 | 17 U |
| Lu864 | ? | QC | wolf | OW |  | Taiga Shield | 676175 | 5949743 | 17 U |
| Lu865 | ? | QC | wolf | OW |  | Taiga Shield | 676175 | 5949743 | 17 U |
| Lu866 | ? | QC | wolf | OW |  | Taiga Shield | 676175 | 5949743 | 17 U |
| Lu868 | ? | QC | wolf | OW |  | Softwood Shield | 700643 | 5410683 | 17 U |
| Lu897 | M | QC | wolf | OW |  | Hudson Plain | 656027 | 5674436 | 17 U |
| Lu900 | F | QC | wolf | OW |  | Softwood Shield | 615224 | 5456454 | 17 U |
| Lu902 | M | QC | wolf | OW |  | Softwood Shield | 712704 | 5383304 | 17 U |
| Lu913 | F | QC | wolf | OW |  | Softwood Shield | 315934 | 5393422 | 18 U |
| Lu922 | M | QC | wolf | OW |  | Softwood Shield | 648224 | 5355279 | 17 U |
| Lu927 | F | QC | wolf | OW |  | Softwood Shield | 648224 | 5355279 | 17 U |
| Lu928 | M | QC | wolf | OW |  | Softwood Shield | 648224 | 5355279 | 17 U |
| Lu1108 | F | QC | wolf | OW |  | Mixed Wood Shield | 478342 | 5178600 | 18 T |
| Lu1132 | F | QC | wolf | OW |  | Softwood Shield | 501177 | 5617947 | 19 U |
| Lu1133 | F | QC | wolf | OW |  | Softwood Shield | 492266 | 5639637 | 19 U |
| Hy617 | ? | QC | hybrid? | NW |  | Softwood Shield | 310313 | 5514163 | 18 U |
| Hy669 | F | QC | hybrid? | NW |  | Mixed Wood Shield | 684791 | 5152421 | 17 T |
| Hy702 | F | QC | hybrid? | NW |  | Softwood Shield | 645449 | 5339637 | 17 U |
| Hy798 | M | QC | hybrid? | NW | NW | Mixed Wood Plains | 278290 | 5140677 | 19 T |
| Hy893 | F | QC | hybrid? | NW |  | Mixed Wood Plains | 518373 | 5007061 | 18 T |
| Hy909 | M | QC | hybrid? | NW | OW | Mixed Wood Shield | 626316 | 5225037 | 17 T |
| Hy941 | M | QC | hybrid? | NW | NW | Atlantic Highlands | 266398 | 5035148 | 19 T |
| co1246 | M | MB | coyote | NW | NW | Temperate Prairies | 408222 | 5664154 | 14 U |
| co1247 | M | MB | coyote | NW | NW | Boreal Plain | 457200 | 5630800 | 14 U |
| co1248 | M | MB | coyote | NW | NW | Boreal Plain | 457200 | 5630800 | 14 U |
| co1249 | M | MB | coyote | NW | NW | Boreal Plain | 457200 | 5630800 | 14 U |
| co1250 | M | MB | coyote | NW | NW | Boreal Plain | 457200 | 5630800 | 14 U |
| co1251 | M | MB | coyote | NW | NW | Boreal Plain | 457200 | 5630800 | 14 U |
| co1252 | M | MB | coyote | NW | NW | Boreal Plain | 457200 | 5630800 | 14 U |
| co1253 | M | MB | coyote | NW |  | Boreal Plain | 457200 | 5630800 | 14 U |
| co1255 | M | MB | coyote | NW | NW | Boreal Plain | 459700 | 5587954 | 14 U |
| co1258 | F | MB | coyote | NW |  | Boreal Plain | 457200 | 5630800 | 14 U |
| co1260 | F | MB | coyote | NW | NW | Temperate Prairies | 466995 | 5605529 | 14 U |
| co1261 | F | MB | coyote | NW |  | Boreal Plain | 459800 | 5587954 | 14 U |
| co1262 | F | MB | coyote | NW |  | Boreal Plain | 459100 | 5587954 | 14 U |
| co1263 | F | MB | coyote | NW |  | Boreal Plain | 457200 | 5630800 | 14 U |
| co1265 | F | MB | coyote | NW |  | Boreal Plain | 459500 | 5587954 | 14 U |
| co1266 | F | MB | coyote | NW | NW | Boreal Plain | 459500 | 5587954 | 14 U |
| co1267 | F | MB | coyote | NW | NW | Boreal Plain | 459400 | 5587954 | 14 U |
| co1269 | M | SK | coyote | NW | NW | Prairies^5^ | 499459 | 5733418 | 13 U |
| co1270 | ? | SK | coyote | NW |  | Prairies^5^ | 499459 | 5733418 | 13 U |
| co1271 | ? | SK | coyote | NW |  | Prairies^5^ | 499459 | 5733418 | 13 U |
| co1273 | M | SK | coyote | NW | NW | Prairies^5^ | 499459 | 5733418 | 13 U |
| co1274 | M | SK | coyote | NW | NW | Prairies^5^ | 499459 | 5733418 | 13 U |
| co1275 | ? | SK | coyote | NW |  | Prairies^5^ | 499459 | 5733418 | 13 U |
| co1276 | ? | SK | coyote | NW |  | Prairies^5^ | 499459 | 5733418 | 13 U |
| co1278 | ? | SK | coyote | NW |  | Prairies^5^ | 499459 | 5733418 | 13 U |
| co1279 | ? | SK | coyote | NW |  | Prairies^5^ | 499459 | 5733418 | 13 U |
| co1280 | ? | SK | coyote | NW |  | Prairies^5^ | 499459 | 5733418 | 13 U |
| co1285 | ? | SK | coyote | NW |  | Prairies^5^ | 499459 | 5733418 | 13 U |
| SK9 | M | SK | coyote | NW | NW | Boreal Plain | 524725 | 5967589 | 13 U |
| co1287 | ? | MB | coyote | NW |  | Boreal Plain | 642104 | 5611009 | 14 U |
| co1288 | ? | MB | coyote | NW |  | Boreal Plain | 651593 | 5539495 | 14 U |
| co1289 | M | MB | coyote | NW | NW | Boreal Plain | 642104 | 5611009 | 14 U |
| lyAVS1 | ? | MB | wolf | NW |  | Boreal Plain | 340000 | 5710000 | 14 U |
| lyAVS9 | F | MB | wolf | NW |  | Temperate Prairies | 515000 | 5605000 | 14 U |
| lyAVS11 | M | MB | wolf | NW | OW | Temperate Prairies | 494142 | 5640561 | 14 U |
| lyAVS27 | F | MB | wolf | NW |  | Boreal Plain | 397718 | 5739835 | 14 U |
| lyAVS28 | F | MB | wolf | NW |  | Boreal Plain | 397718 | 5739835 | 14 U |
| lyAVS30 | F | MB | wolf | NW |  | Boreal Plain | 370000 | 5755000 | 14 U |
| lyAVS50 | M | MB | wolf | NW |  | Boreal Plain | 395000 | 5735000 | 14 U |
| lyAVS52 | F | MB | wolf | NW |  | Boreal Plain | 340000 | 5695000 | 14 U |
| lyAVS61 | F | MB | wolf | NW |  | Boreal Plain | 339000 | 5741000 | 14 U |
| lyAVS62 | F | MB | wolf | NW | OW | Temperate Prairies | 465104 | 5677745 | 14 U |
| ly1223 | M | MB | wolf | NW |  | Boreal Plain | 325112 | 5631500 | 14 U |
| SK1 | ? | SK | wolf | NW |  | Boreal Plain | 312932 | 5967368 | 13 U |
| SK2 | M | SK | wolf | NW | NW | Boreal Plain | 312932 | 5967368 | 13 U |
| SK3 | ? | SK | wolf | NW |  | Boreal Plain | 312932 | 5967368 | 13 U |
| SK4 | M | SK | wolf | NW | NW | Boreal Plain | 524725 | 5967589 | 13 U |
| SK5 | M | SK | wolf | NW | NW | Boreal Plain | 312932 | 5967368 | 13 U |
| SK6 | ? | SK | wolf | NW |  | Boreal Plain | 312932 | 5967368 | 13 U |
| SK7 | M | SK | wolf | NW | OW | Boreal Plain | 312932 | 5967368 | 13 U |
| luAVS3 | M | MB | wolf | OW | OW | Boreal Plain | 662259 | 5661645 | 14 U |
| luAVS4 | F | MB | wolf | OW |  | Temperate Prairies | 410000 | 5433000 | 14 U |
| luAVS10 | M | MB | wolf | OW | OW | Boreal Plain | 459545 | 5588000 | 14 U |
| luAVS13 | M | MB | wolf | OW | OW | Temperate Prairies | 485100 | 5631600 | 14 U |
| luAVS15 | M | MB | wolf | OW | OW | Boreal Plain | 409010 | 5629042 | 14 U |
| luAVS18 | M | MB | wolf | OW |  | Boreal Plain | 373000 | 5615000 | 14 U |
| luAVS19 | M | MB | wolf | OW | NW | Temperate Prairies | 389000 | 5602000 | 14 U |
| luAVS23 | F | MB | wolf | OW |  | Boreal Plain | 368312 | 5741322 | 14 U |
| luAVS25 | M | MB | wolf | OW | OW | Boreal Plain | 357139 | 5846562 | 14 U |
| luAVS26 | M | MB | wolf | OW | OW | Boreal Plain | 397718 | 5739835 | 14 U |
| luAVS29 | F | MB | wolf | OW | OW | Boreal Plain | 357139 | 5846562 | 14 U |
| luAVS31 | F | MB | wolf | OW |  | Boreal Plain | 361981 | 5771201 | 14 U |
| luAVS32 | M | MB | wolf | OW | OW | Temperate Prairies | 465000 | 5625000 | 14 U |
| luAVS33 | M | MB | wolf | OW | OW | Boreal plain | 428500 | 5653000 | 14 U |
| luAVS34 | F | MB | wolf | OW |  | Boreal Plain | 360000 | 5720000 | 14 U |
| luAVS40 | F | MB | wolf | OW | OW | Temperate Prairies | 468254 | 5622121 | 14 U |
| luAVS41 | F | MB | wolf | OW |  | Temperate Prairies | 460900 | 5616600 | 14 U |
| luAVS42 | M | MB | wolf | OW | OW | Boreal Plain | 334500 | 5863000 | 14 U |
| luAVS43 | M | MB | wolf | OW | OW | Boreal Plain | 377779 | 5649544 | 14 U |
| luAVS44 | M | MB | wolf | OW | OW | Boreal Plain | 430000 | 5590000 | 14 U |
| luAVS45 | M | MB | wolf | OW | OW | Temperate Prairies | 456535 | 5630960 | 14 U |
| luAVS47 | M | MB | wolf | OW | OW | Boreal Plain | 458586 | 5588828 | 14 U |
| luAVS48 | F | MB | wolf | OW |  | Boreal Plain | 418137 | 5650473 | 14 U |
| luAVS49 | M | MB | wolf | OW | OW | Softwood Shield | 346000 | 6111000 | 14 U |
| luAVS51 | M | MB | wolf | OW | OW | Boreal Plain | 334500 | 5863000 | 14 U |
| luAVS54 | M | MB | wolf | OW | OW | Boreal Plain | 376330 | 5649754 | 14 U |
| luAVS55 | F | MB | wolf | OW |  | Boreal Plain | 370000 | 5640000 | 14 U |
| luAVS56 | M | MB | wolf | OW | OW | Boreal Plain | 357000 | 5820800 | 14 U |
| luAVS57 | ? | MB | wolf | OW |  | Boreal Plain | 357000 | 5820800 | 14 U |
| luAVS59 | M | MB | wolf | OW |  | Boreal Plain | 395000 | 5734000 | 14 U |
| luAVS60 | M | MB | wolf | OW | OW | Boreal Plain | 356000 | 5705000 | 14 U |
| luAVS63 | F | MB | wolf | OW |  | Boreal Plain | 339000 | 5741000 | 14 U |
| luAVS64 | M | MB | wolf | OW | OW | Boreal Plain | 465484 | 5733351 | 14 U |
| luAVS65 | M | MB | wolf | OW | OW | Temperate Prairies | 468254 | 5622121 | 14 U |
| luAVS69 | F | MB | wolf | OW |  | Boreal Plain | 353000 | 5655500 | 14 U |
| luAVS70 | M | MB | wolf | OW |  | Boreal Plain | 353000 | 5655500 | 14 U |
| lu1200 | M | MB | wolf | OW |  | Temperate Prairies | 493800 | 5508900 | 14 U |
| lu1201 | M | MB | wolf | OW | OW | Temperate Prairies | 493800 | 5508900 | 14 U |
| lu1204 | F | MB | wolf | OW |  | Temperate Prairies | 493800 | 5508900 | 14 U |
| lu1205 | M | MB | wolf | OW |  | Temperate Prairies | 493800 | 5508900 | 14 U |
| lu1207 | M | MB | wolf | OW |  | Boreal Plain | 395621 | 5620559 | 14 U |
| lu1208 | F | MB | wolf | OW |  | Boreal Plain | 370000 | 5640000 | 14 U |
| lu1209 | F | MB | wolf | OW |  | Boreal Plain | 456097 | 5625368 | 14 U |
| lu1211 | M | MB | wolf | OW | OW | Boreal Plain | 449250 | 5599500 | 14 U |
| lu1212 | F | MB | wolf | OW |  | Boreal Plain | 351850 | 5654744 | 14 U |
| lu1214 | F | MB | wolf | OW |  | Boreal Plain | 351850 | 5654744 | 14 U |
| lu1216 | F | MB | wolf | OW |  | Boreal Plain | 455705 | 5625501 | 14 U |
| lu1218 | M | MB | wolf | OW |  | Boreal Plain | 443301 | 5639933 | 14 U |
| lu1219 | F | MB | wolf | OW |  | Boreal Plain | 443660 | 5605000 | 14 U |
| lu1220 | M | MB | wolf | OW |  | Boreal Plain | 443660 | 5604900 | 14 U |
| lu1221 | M | MB | wolf | OW |  | Boreal Plain | 375212 | 5650572 | 14 U |
| lu1245 | M | MB | wolf | OW | OW | Boreal Plain | 403666 | 5631654 | 14 U |
| SK8 | ? | SK | wolf | OW |  | Boreal Plain | 312932 | 5967368 | 13 U |
| SK10 | ? | SK | wolf | OW |  | Boreal Plain | 524725 | 5967589 | 13 U |
| LU1231 | M | SK | wolf | OW |  | Boreal Plain | 580923 | 6023067 | 12 U |
| LU1233 | F | SK | wolf | OW |  | Boreal Plain | 333523 | 6114042 | 13 U |
| LU1234 | M | SK | wolf | OW | OW | Boreal Plain | 580923 | 6023067 | 12 U |
| LU1235 | F | SK | wolf | OW |  | Boreal Plain | 657490 | 6192693 | 12 U |
| LUAVS22 | M | SK | wolf | OW | OW | Boreal Shield | 670748 | 6228034 | 13 V |
| H1286 | M | MB | hybrid? | NW | OW | Boreal Plain | 651593 | 5543495 | 14 U |
| lU16 | ? | AB | wolf | OW |  | Unknown | Unknown | Unknown | Unkn. |
| lU1224 | ? | YK | wolf | OW |  | Boreal Cordillera | 576588 | 7104922 | 7 W |
| lU1225 | M | AB | wolf | OW | OW | Boreal Plain | 435094 | 6119937 | 11 U |
| lU1226 | ? | BC | wolf | OW |  | Western Cordillera | 648978 | 5542988 | 11 U |
| lU1227 | ? | BC | wolf | OW |  | Boreal Plain | 680035 | 6178175 | 10 U |
| lU1228 | M | BC | wolf | OW |  | Western Cordillera | 433892 | 5985600 | 10 U |
| W013 | F | ON | wolf | NW |  | Mixed Wood Shield | 712091 | 5046018 | 17 T |
| W022 | F | ON | wolf | NW |  | Mixed Wood Shield | 678627 | 5038024 | 17 T |
| W065 | M | ON | wolf | NW | NW | Mixed Wood Shield | 268097 | 5096513 | 18 T |
| W075 | M | ON | wolf | NW |  | Mixed Wood Shield | 709962 | 5075331 | 17 T |
| W090 | M | ON | wolf | NW | NW | Mixed Wood Shield | 669222 | 5041760 | 17 T |
| W091 | M | ON | wolf | NW | NW | Mixed Wood Shield | 291555 | 5071451 | 18 T |
| W167 | M | ON | wolf | NW | NW | Mixed Wood Shield | 278929 | 5047252 | 18 T |
| Wsun | F | ON | wolf | NW |  | Mixed Wood Shield | 702747 | 5050637 | 17 T |
| NEON1 | F | ON | wolf | OW |  | Softwood Shield | 447751 | 5495886 | 17 U |
| NEON2 | M | ON | wolf | OW | OW | Softwood Shield | 440597 | 5495960 | 17 U |
| NEON3 | F | ON | wolf | OW |  | Softwood Shield | 530858 | 5455672 | 17 U |
| NEON4 | M | ON | wolf | NW | OW | Softwood Shield | 535848 | 5460739 | 17 U |
| NEON5 | M | ON | wolf | NW | OW | Softwood Shield | 520201 | 5460645 | 17 U |
| NEON6 | F | ON | wolf | OW |  | Softwood Shield | 520111 | 5451630 | 17 U |

^1^AB=Alberta, BC=British Columbia, MB=Manitoba, ON=Ontario, QC=Québec, SK=Saskatchewan, YK=Yukon Territory

^2^NW= New World (North American) mtDNA, OW= Old World (Eurasian) mtDNA.

^3^Y-chromosome marker MS41A with allele 208 originating from OW canids and other alleles (212-216) from NW canids (Hailer and Leonard 2008)

^4^United States Environmental Protection Agency (2006)

^5^West-Central Semi-Arid Prairies

Literature cited:

Hailer F, Leonard JA (2008) Hybridization among three native North American *Canis* species in a region of natural sympatry. PLoS ONE 3:e3333

United States Environmental Protection Agency (2006) Level III Ecoregions of North America (http://www.epa.gov/wed/pages/ecoregions/na_eco.htm#Level%20III, accessed May 2011)

Table S2. Mitochondrial DNA (mtDNA) control-region haplotypes in wolves and coyotes from Saskatchewan and Québec, Canada. Sample locations are provided in Table S1.

| **Sample** | **Sex** | **Province^1^** | **Morphology** | **mtDNA haplotype** | **GenBank^2^ accession no.** |
| --- | --- | --- | --- | --- | --- |
| Co618 | F | QC | coyote | C19 | AY267736 |
| Co638 | M | QC | coyote | la18^3^ | GQ849372 |
| Co647 | M | QC | coyote | C1 | AY267718 |
| Co648 | F | QC | coyote | la18^3^ | GQ849372 |
| Co732 | M | QC | coyote | C19 | AY267736 |
| Ly875 | F | QC | wolf | C14 | AY267731 |
| Ly876 | M | QC | wolf | C14 | AY267731 |
| Ly914 | F | QC | wolf | C14 | AY267731 |
| Ly1134 | M | QC | wolf | C19 | AY267736 |
| Lu734 | M | QC | wolf | C22 | FJ687608 |
| Lu746 | M | QC | wolf | C22 | FJ687608 |
| Lu831 | M | QC | wolf | C22 | FJ687608 |
| Lu1132 | F | QC | wolf | C22 | FJ687608 |
| Lu1133 | F | QC | wolf | C22 | FJ687608 |
| SK9 | M | SK | coyote | la31^4^ | FM209399 |
| SK1 | ? | SK | wolf | C3 | AY267720 |
| SK2 | M | SK | wolf | C3 | AY267720 |
| SK3 | ? | SK | wolf | C3 | AY267720 |
| SK4 | M | SK | wolf | C3 | AY267720 |
| SK5 | M | SK | wolf | C3 | AY267720 |
| SK6 | ? | SK | wolf | C3 | AY267720 |
| SK7 | M | SK | wolf | C13 | AY267730 |
| SK8 | ? | SK | wolf | 16 | HM014466 |
| SK10 | ? | SK | wolf | 16 | HM014466 |

^1^QC=Québec and SK=Saskatchewan

^2^http://www.ncbi.nlm.nih.gov/genbank/

^3^Sequence shorter than la18 but identical starting from base pair 18

^4^Sequence shorter than la31 but identical starting from base pair 42


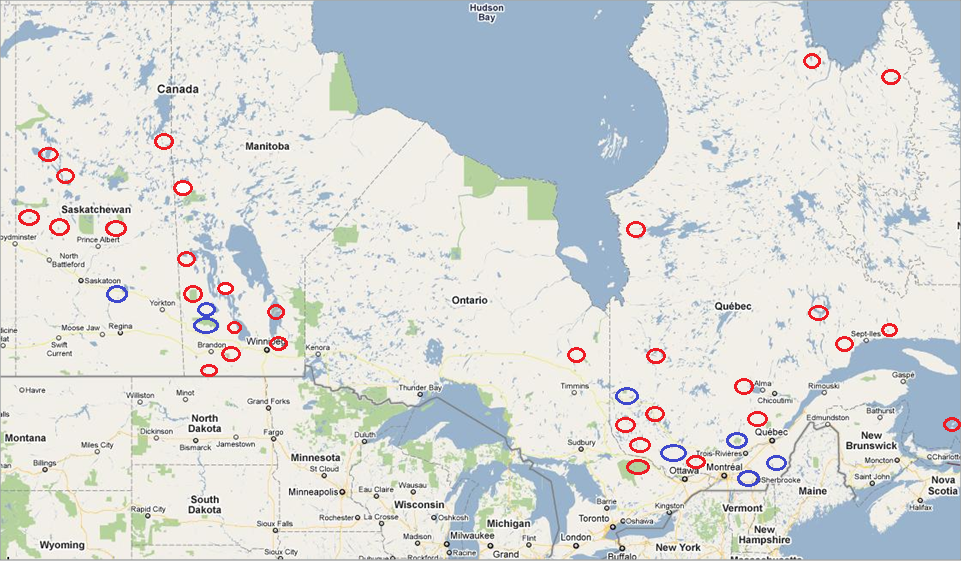


**2**

**3**

**1**

Figure S1. Map of primary sampling locations for the Prairies (Manitoba and Saskatchewan) and Québec, Canada. Ontario reference samples are shown, but six reference samples from Western Canada (British Columbia, Alberta, and Yukon Territory) are not included. Areas where 1-10 samples were collected are marked in red, and areas where 11-50 samples were collected are marked in blue. UTM locations and identification of canids are provided in Table S1. Wolves with NW mtDNA haplotypes extended eastward to Manicouagan Reservoir, Québec (1) and westward to Chitek Lake, Saskatchewan (2). Manitoba wolves with NW mtDNA were located in or near Duck Mountain Provincial Park and Forest (3).

Table S3. Probability values for the number of genetic clusters (L(K) with standard deviation (SD) and ΔK) for wolves and coyotes from the Prairies and Québec, Canada, using K=1 – 10.

| **K** | **L(K)** | **SD** | **ΔK** |
| --- | --- | --- | --- |
| 1 | -10899.3 | 0.230 | - |
| 2 | -10403.5 | 0.358 | 803.140 |
| 3 | -10195.2 | 0.834 | 239.986 |
| 4 | -10186.6 | 20.906 | 7.086 |
| 5 | -10029.8 | 31.209 | 5.143 |
| 6 | -10033.6 | 225.259 | 0.610 |
| 7 | -9899.9 | 10.682 | 7.748 |
| 8 | -9849.0 | 9.210 | 2.927 |
| 9 | -9825.1 | 10.557 | 1.635 |
| 10 | -9783.9 | 0.000 | - |


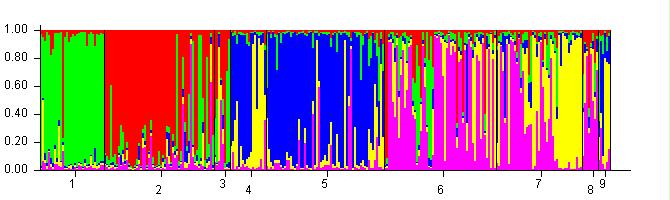


--coPR----------coQC--------h woPR-----woPR-OW---------woQC-NW------woQC-O---ALW

-NW

Figure S2. Assignment values for Prairie coyotes (coPR), Québec coyotes (coQC), canids morphologically classified as possible hybrids (h), Prairie wolves (woPR) and Québec wolves (woQC) according to STRUCTURE results for K=5 clusters. Wolves are classified as having New World (NW) or Old World (OW) mtDNA. Samples from Algonquin Provincial Park (AL) in Ontario and samples from Western Canada (W) originating from British Columbia, Alberta, and Yukon Territory were included for reference.


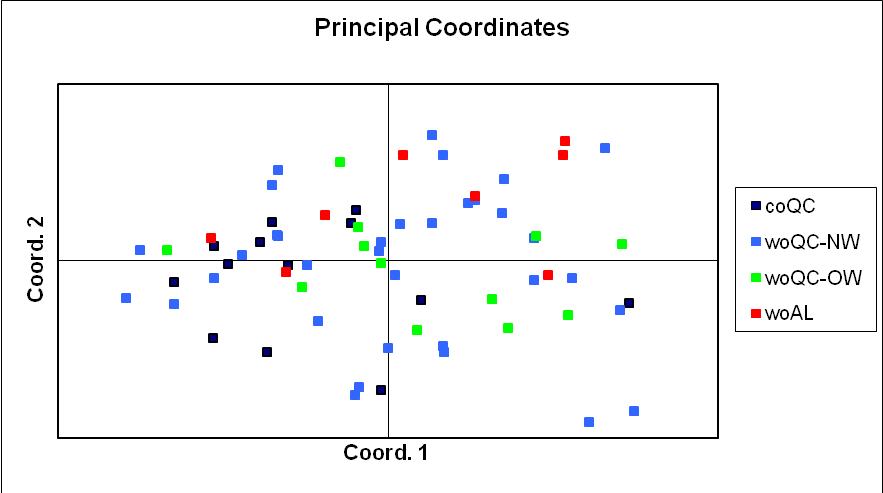


Figure S3. Principal coordinate analysis for Québec coyotes (coQC, n=13), wolves with New World (woQC-NW, n=34) and Old World (woQC-OW, n=12) mtDNA, and wolves from Algonquin Provincial Park, Ontario (n=8) sampled in the Mixed Wood Shield ecoregion. The 1^st^ and 2^nd^ axes explained 22.0% and 19.1% of the variation, respectively.


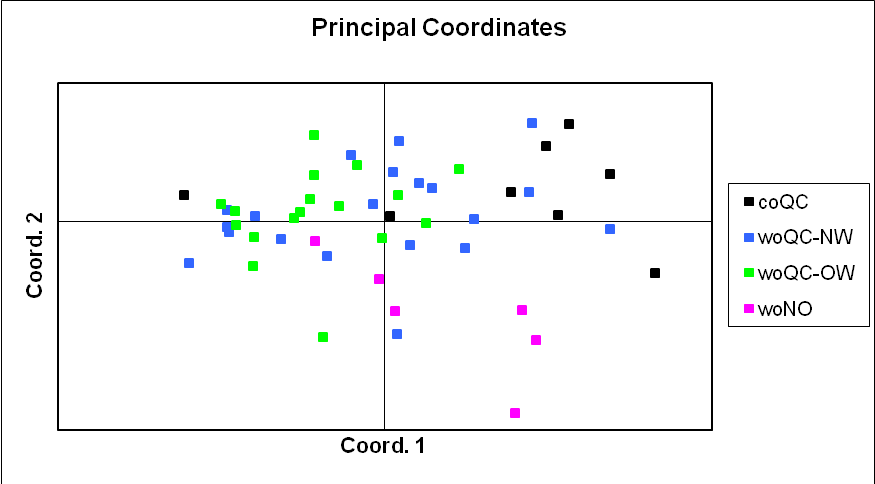


Figure S4. Principal coordinate analysis for Québec coyotes (coQC, n=8), wolves with New World (woQC-NW, n=20) and Old World (woQC-OW, n=17) mtDNA, and wolves from northeastern Ontario (n=6) sampled in the Softwood Shield ecoregion. The 1^st^ and 2^nd^ axes explained 23.3% and 18.9% of the variation, respectively.


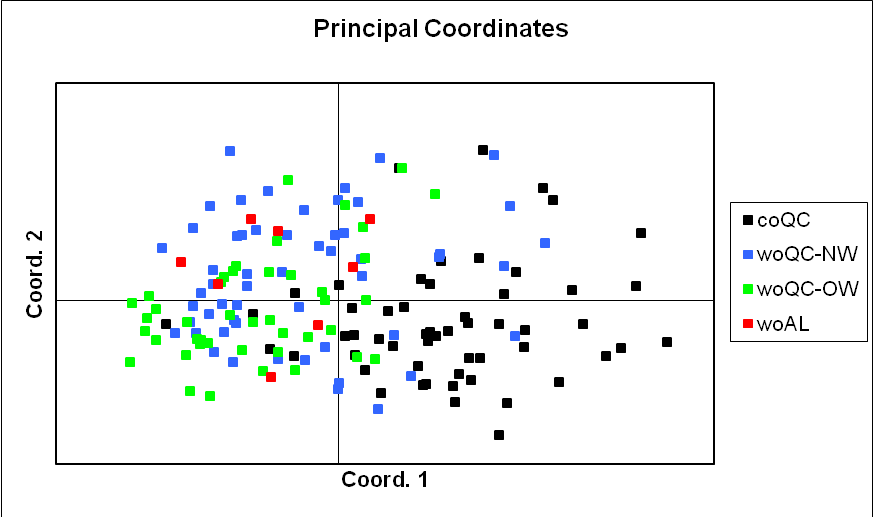


Figure S5. Principal coordinate analysis for Québec coyotes (coQC, n=55), wolves with New World (woQC-NW, n=56) and Old World (woQC-OW, n=43) mtDNA, and wolves from Algonquin Provincial Park, Ontario (n=8). The 1^st^ and 2^nd^ axes explained 25.6% and 19.3% of the variation, respectively.
